# Sexual dimorphism in the incidence of human cancers

**DOI:** 10.1186/s12885-019-5902-z

**Published:** 2019-07-12

**Authors:** Daoshan Zheng, Justyna Trynda, Cecilia Williams, Jeremy A. Vold, Justin H. Nguyen, Denise M. Harnois, Sanjay P. Bagaria, Sarah A. McLaughlin, Zhaoyu Li

**Affiliations:** 1 Department of Cancer Biology, Mayo Clinic, 4500 San Pablo Road, Griffin 210, Jacksonville, FL 32224 USA; 2 KTH Royal Institute of Technology, Karolinska Institutet, Science for Life Laboratory, Stockholm, Sweden; 3 Mayo Cancer Registry, Mayo Clinic, 4500 San Pablo Road, Jacksonville, FL 32224 USA; 4 Department of Surgery and Mayo Clinic Cancer Center, Mayo Clinic, 4500 San Pablo Road, Jacksonville, FL 32224 USA

**Keywords:** Sexual dimorphism, Cancer incidence, Human cancers

## Abstract

**Background:**

Sex differences in the incidences of cancers become a critical issue in both cancer research and the development of precision medicine. However, details in these differences have not been well reported. We provide a comprehensive analysis of sexual dimorphism in human cancers.

**Methods:**

We analyzed four sets of cancer incidence data from the SEER (USA, 1975–2015), from the Cancer Registry at Mayo Clinic (1970–2015), from Sweden (1970–2015), and from the World Cancer Report in 2012.

**Results:**

We found that all human cancers had statistically significant sexual dimorphism with male dominance in the United States and mostly significant in the Mayo Clinic, Sweden, and the world data, except for thyroid cancer, which is female-dominant.

**Conclusions:**

Sexual dimorphism is a clear but mostly neglected phenotype for most human cancers regarding the clinical practice of cancer. We expect that our study will facilitate the mechanistic studies of sexual dimorphism in human cancers. We believe that fully addressing the mechanisms of sexual dimorphism in human cancers will greatly benefit current development of individualized precision medicine beginning from the sex-specific diagnosis, prognosis, and treatment.

**Electronic supplementary material:**

The online version of this article (10.1186/s12885-019-5902-z) contains supplementary material, which is available to authorized users.

## Background

Sex dimorphism is a critical phenotype of human cancers, however, the investigation of such important topic has been barely conducted, and most importantly, sex-specific clinical diagnosis and treatment of human cancers has been mostly overlooked. Sexual dimorphism of human cancers is also poorly understood with regards to etiology and prevention. Although hundreds of cancer epidemiological reports are published each year, these reports primarily focus on geographic locations of specific countries, regions, or worldwide, specific cancer types, and/or specific populations [1–19]. Cancer caused about one-fourth of the deaths in the United States each year [20] and is a major public health issues in the United States and worldwide. Multiple factors could contribute to sex dimorphism of human cancers, such as sex-specific genetic variations and mutations and sex-specific responses to carcinogens [21–23]. The important variable of sex has been greatly underestimated for its impact on cancer initiation and progression, and therefore its translational application has been barely conducted. To deliver a comprehensive study on the sexual dimorphism in each cancer type, we investigated the latest Surveillance, Epidemiology, and End Results Program (SEER) data (1975–2015) from the National Cancer Institute (NCI) in the USA, the Cancer Registry data at Mayo Clinic (1970–2015), the cancer incidence data of Sweden (1970–2015), and the World Cancer Report data in 2012 to address the significance of sexual dimorphism in each human cancer type or subtype. Our definition of sexual dimorphism in each cancer was based on the calculation of the significance of cancer incidence between men and women during the past 40 years (*p* values less than 0.05). Thus, we provide a comprehensive overview about sex differences in the cancer incidence worldwide, which will be critical for both basic cancer research and translational application, i.e., guiding the mechanistic studies of sex dimorphism in human cancers and developing sex-specific cancer precision medicine.

## Methods

### Sources of cancer incidence data

The incidence data for all types of cancers were collected from four sources, the SEER Program (NCI/NIH), the cancer incidence data of Sweden (Swedish Cancer Registry), the Cancer Registry data from Mayo Clinic, and the World Cancer Report data in 2012 (IARC). The SEER 9 data cover age-adjusted cancer incidence rates for 1975–2015 among the US population. The Swedish Cancer Registry, founded in 1958, covers age-adjusted cancer incidence rates for the whole Swedish population from 1970 to 2015 and the completeness is estimated to be 96% [24]. The Cancer Registry data from Mayo Clinic data include all clinical records of cancer patients at Mayo Clinic Hospitals for all three sites in the country, Rochester (MN), Scottsdale (AZ), and Jacksonville (FL), in 1970–2015. The world cancer statistics data in 2012 were collected from the Cancer Incidence in Five Continents (CI5) and GLOBOCAN, and the ratios of the incidence rates between men and women were calculated.

### Data analysis and statistical methods

#### Incidence rates

We collected the crude incidence rate for 30 types or subtypes of human cancers from the SEER data and Mayo Clinic, 29 types or subtypes of human cancers from Sweden data, and 24 types from the World Cancer Report in 2012. All these incidence rates were standardized to the population of 100,000 and were age-adjusted to the World standard population (WHO 2000–2025) as the age-adjusted standardized rate (ASR). The incidence rate for men and women were analyzed for each year between 1975 and 2015 for the SEER data, between 1970 and 2015 for the Swedish data, and in 2012 for the World Cancer Report data. For the Mayo Clinic data between 1970 and 2015, we had the case numbers of male and female patients but not the incidence rates.

#### Sex-dimorphic incidence ratio

For the SEER and Sweden data, the annual sex-dimorphic incidence ratios were calculated from yearly male and female incidence rates as well as per the average ratios for the entire analysis period as the final incidence ratios of men to women for each cancer type. For the Mayo Clinic data between 1975 and 2015, the annual sex-dimorphic incidence ratios were calculated from the case numbers between men and women in each year as well as per the average ratios for the entire analysis period for each cancer type. For the World Cancer Report data, the age-standardized rate (ASR) ratios between men and women were calculated as the cancer incidence ratios between men and women. If the incidence ratio was less than 1, the negative reciprocal ratio was used as the sex-dimorphic ratio.

#### Statistical analysis

The Gamma Distribution test, Poisson distribution test, ANOVA test, and Student’s t-test were performed to calculate the significance of the incidence rates between men and women, and the *p*-value less than 0.05 was considered as statistically significant.

## Results

### The overview of sex differences in the incidence of human cancers

**Fig. 1:**
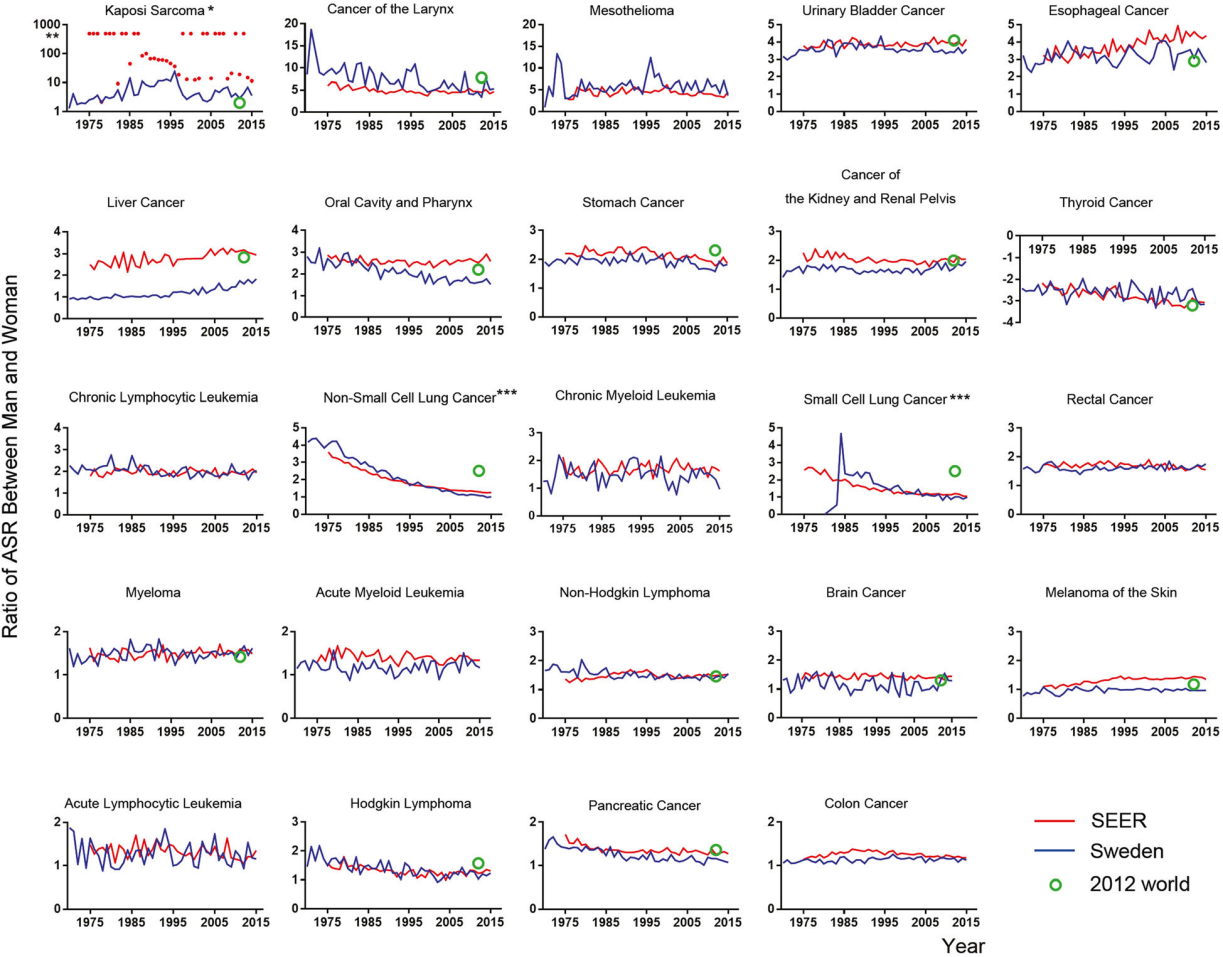
The yearly incidence ratios of men to women in the SEER data (red line), the Swedish data (blue line), and the World Cancer Report 2012 data (green circle). *, the incidence ratios were infinite because of no incidence in women for certain years

**Fig. 2:**
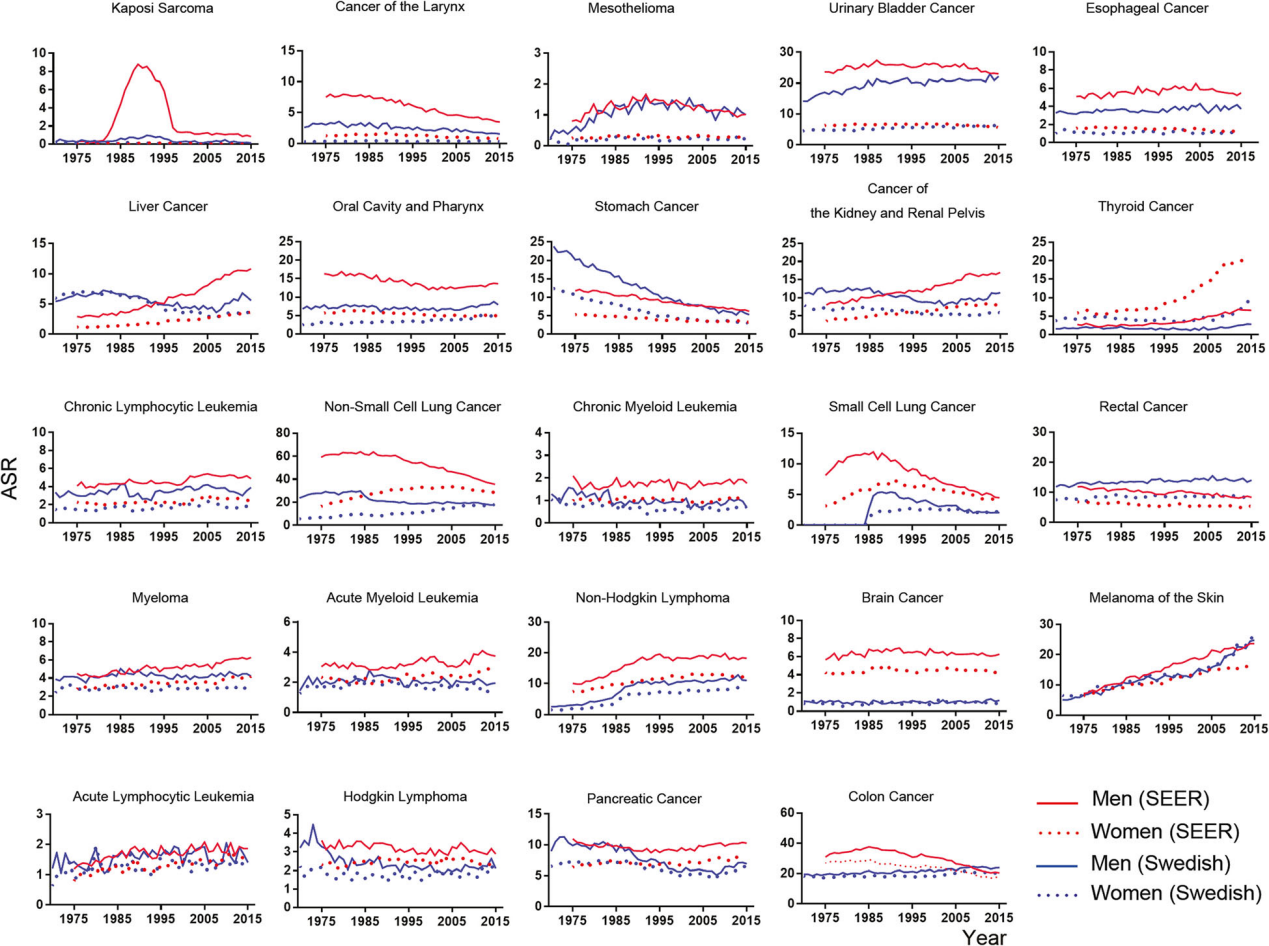
The yearly SEER (red) and Sweden (blue) age-adjusted incidence rates of sex-dimorphic cancers in 1975–2015. The age-adjusted standardized incidence rates (ASR) are per 100,000 and are age-adjusted to the World Standard population. Men, solid line; women; dot line

Based on organ specificities between sexes, we categorized human cancers into two groups: sex-dimorphic and sex-specific (which is also sex-dimorphic but only presented in one gender, including male- and female-specific) cancers. We investigated total 30 types of human cancers (Figs. 1 and 2, Table 1, and Additional file 1: Table S1-S3 and Figure S1-S2) and found that 24 of them were sex-dimorphic with statistical significance, two of them were men-specific (prostate cancer and testicular cancer), and four of them were women-specific (breast cancer, cancer of the cervix uteri, cancer of the corpus and uterus, NOS (not otherwise specified), and ovarian cancer). Breast cancer has extremely low incidence in men [25, 26], but we still considered breast cancer as female-specific because mammary glands are barely developed in men. Except for thyroid cancer, which is female-dominant, the rest of 23 types of sex-dimorphic cancers are all male-dominant.

**Table 1:**
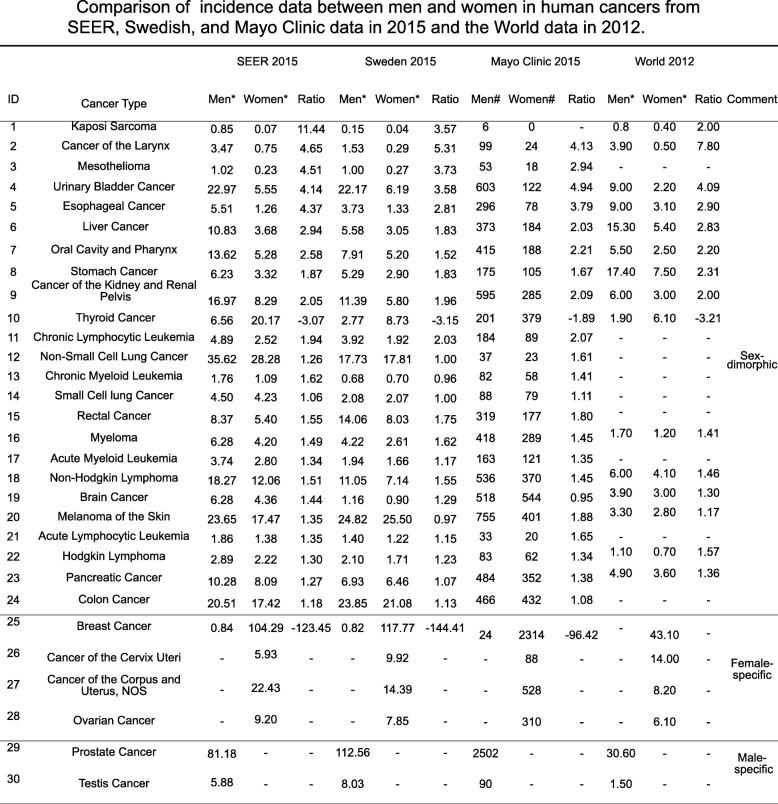
Comparison of incidence data between men and women in human cancers from SEER, Swedish, and Mayo Clinic data in 2015 and the World data in 2012

*, The incidence rates are per 100,000 and are age-adjusted to the World Standard population (WHO 2000–2025). #, The cancer registry cases from Mayo Clinic include all clinical records of cancer patients at Mayo Clinic Hospitals for all three sites in the country in 2015, Rochester, MN, Scottsdale, AZ, and Jacksonville, FL. Ratio, men/women. -, data not available

Overall, sexual dimorphism of human cancers in different datasets were highly consistent, but some notable differences were observed; e.g., in the SEER data sets all 24 cancer types showed sex-dimorphic properties (Figs. 1 and 2 and Additional file 1: Table S1), while in the Mayo Clinic data the incidence ratios for non-hodgin lymphoma, brain cancer, and pancreatic cancer were not significantly different between the sexes (Additional file 1: Table S3 and Figure S1 and S2), and in the Swedish dataset, liver cancer and melanoma of the skin showed no significant incidence differences between men and women (Fig. 1, and Additional file 1: Table S2). From the World Cancer Report data in 2012 (Fig. 1 and Table 1), we found that the incidence ratios between men and women in 10 out of total 24 cancer types were over 2-fold, including thyroid cancer, still the only female-dominant sex-dimorphic cancer. Thus, these sex-dimorphic data are mostly consistent among the different datasets, indicating that sexual dimorphism in human cancers is persistent and similar across the USA, Sweden, and worldwide.

### Geographical differences in sexual dimorphism of human cancers

We compared sexual dimorphism in human cancer incidences between two countries, USA and Sweden, for two aspects: 1) differences in the sexual incidence ratios; 2) differences in the individual cancer incidence. For the former, most cancer types showed no significant differences in the sexual incidence ratios between USA and Sweden (Fig. 1); strikingly, both liver cancer and skin cancer showed substantially higher sexual incidence ratios across the past 50 years in USA but not in Sweden, even though Sweden had relatively higher incidence for both caners than USA (Fig. 1); both USA and Sweden had similar sexual dimorphism of oral cancer and esophageal cancer in the early years but USA showed mildly higher sexual dimorphism of both cancers in recent two decades (Fig. 1); both USA and Sweden had recent burst of thyroid cancer in a recent decade but with increased sexual dimorphism in USA (Figs. 1 and 2); Sweden only had higher sexual incidence ratios in lung cancer for the previous three decades (1970s–1990s) than USA but the ratios became identical in the past decade (Fig. 1). For the latter, USA has higher incidences in mesothelioma, urinary bladder cancer, melanoma of the skin, and colorectal cancer than Sweden (Fig. 2); Sweden has higher incidences in oral cancer, chronic myeloid leukemia, brain cancer, and myeloma than USA (Fig. 2).

### Historical changes of sexual dimorphism of human cancers

Sexual dimorphism of human cancer incidence rates has been quite steady for most of human cancers throughout the past five decades (Fig. 1). In USA, significantly increases in sexual dimorphism of human cancers were only observed in skin cancer, esophageal cancer, and thyroid cancer in recent decades (Fig. 1). Sexual dimorphism of in small and non-small cell lung cancer was constantly reduced throughout the past five decades in both USA and Sweden and the reduction of sexual dimorphism of oral cancer was only observed in Sweden but not in USA, which were due to increased female incidence and/or reduced male incidence (Figs. 1 and 2). Upon successful HIV treatments, reduced incidence of Kaposi sarcoma was observed in both USA and Sweden in the past two decades but the changes of sexual dimorphism was not clear because of lack of female incidence for certain years (Figs. 1 and 2). In Sweden, although the incidence of melanoma of the skin continuously and dramatically increased up to at least 6-fold in the past 50 years, no sexual dimorphism was observed (Fig. 1). The incidence rates of larynx cancer and kidney cancer in USA catch those in Sweden in the recent years whereas the incidence rates of non-Hodgkin lymphoma and pancreatic cancer in Sweden catch those in USA recently (Fig. 2). The incidence of stomach cancer continuously drops in both USA and Sweden throughout years but no change was observed in sexual dimorphism (Figs. 1 and 2). Both USA and Sweden have dramatic increase in the incidence of non-Hodgkin lymphoma since around 1985 but the sexual incidence ratios remain the same (Figs. 1 and 2). Therefore, historically there were many differences in cancer incidence and sexual dimorphism of incidence between USA and Sweden.

### Most recent status of sexual dimorphism in human cancers

We summarized the most recent status of sexual dimorphism in human cancers from the SEER/USA (2015), Sweden (2015), Mayo Clinic (2015), and World (2012) cancer data (Table 1) because these are the immediate questions to be answered at present. Sexual dimorphism of most cancer types showed no significant difference among four datasets, especially between SERR and Mayo data. The major differences between SEER and Mayo data were that Mayo had less sexual dimorphism in stomach cancer and thyroid cancer. The major differences in sexual dimorphism were observed in Kaposi sarcoma, laryngeal cancer, esophageal cancer, and skin cancer among USA, Swedish, and worldwide data (Table 1); USA showed much higher sexual dimorphism in Kaposi sarcoma, esophageal cancer, and skin cancer and less sexual dimorphism in laryngeal cancer than Sweden and worldwide. Most differences in sexual dimorphism of cancers were observed between USA and Sweden; USA had higher sexual dimorphism in liver caner and oral cancer but less sexual dimorphism in mesothelioma than Sweden. These most recent data provide invaluable evidence to support our sexual dimorphism studies.

### Sexual dimorphism between Mayo Clinic hospitalization and the USA populations

It is not surprising that sexual dimorphism of most cancers were similar between Mayo Clinic and SEER data because our Mayo Clinic data were collected from three major sites, Rochester (MN), Scottsdale (AZ), and Jacksonville (FL), in the country (Table 1), indicating that the visit of Mayo Clinic hospitals from cancer patients represent the incidence trend of human cancers in the entire US populations. Strikingly, however, the patient numbers of many cancers substantially and constantly increased throughout the past five decades, including urinary bladder cancer, esophageal cancer, liver cancer, oral cancer, kidney cancer, thyroid cancer, leukemia, myeloma, lymphoma, brain cancer, skin cancer, pancreatic cancer, and colorectal cancer, even though the incidences of these cancers in USA did not show significant changes (Fig. 2 and Additional file 1: Figure S2). A few inconsistences of sex-dimorphic ratios between SEER and Mayo Clinic data will raise our cautions when we choose the tissue samples from Mayo Clinic cancer patients for sexual dimorphism studies (Additional file 1: Figure S2). Although more detailed reasons would be interesting to investigate, improved treatments, such as new drugs and novel immunotherapies, might have major contributions for these bursts.

## Discussion

Sex is one of the most obvious features or variables in human beings or mammals. Sex differences in the susceptibility of human cancers were discovered almost a century ago. However, the mechanisms underlying sexual dimorphism in human cancers have been under-investigated and therefore related clinical applications have been barely conducted. In addition to genetic variations and environmental exposures [21–23], many other factors, such as life styles, and behaviors, could play important roles in sex differences of cancer incidences [27–38], e.g., previous studies also showed that sex-dimorphic energy balance and homeostasis might lead to sex differences in gastrointestinal cancers [38], miRNA expression were also sex-dimorphic in many types of cancers [39, 40], non-mining men still had higher incidence of mesothelioma than non-mining women [27–30], and male-dominant HIV infection did not show similar degrees of male-dominant incidence in Kaposi Sarcoma [32–37]. However, sex hormones are the natural differences between males and females, and most sex-dimorphic factors could lead to the changes in the levels of sex hormones or most sex-dimorphic observations could be derived from the differences in sex hormone signaling between sexes. Sex hormones, i.e., estrogens in women and androgens in men, are the drivers of sexual dimorphism and their signaling through estrogen receptors (ERα, ERβ, and/or GPER1) and androgen receptor (AR). However, the mechanisms underlying the regulation of these sex hormone receptors in sex disparities of most human cancers are still poorly understood, except for liver cancer with better understanding from our recent studies [41, 42]. We summarized at least four major challenges in addressing the mechanisms underlying sexual dimorphism in human cancers include: 1) various degrees of sex hormone signaling due to sex hormone receptor expression, different tissues, and tumor development stages; 2) under-characterized specificities of antibodies used for measuring sex hormone receptors [43]; 3) lack of genetic assays od sex hormone receptors with clear knockouts in vivo using transgenic mice or CRISPR/Cas9; and 4) multiple unclear risk factors that could contribute to sexual dimorphism of human cancers [27–37]. Conquering these challenges will greatly improve our understanding of the mechanisms underlying sexual dimorphism in human cancers; i.e., why most of human cancers are males-dominant? Why sex hormone levels change in different ages? And whether there is a general rule to control sex-dimorphic regulation in human cancers?

Sex differences in the incidences of cancers become a critical issue in both cancer research and the development of precision medicine. Following the increasing understanding of sexual dimorphism in the incidence of these cancers, sex-specific diagnosis, prognosis, and treatment would become an important addition and an initial step towards personalized precision medicine.

The interesting regional differences in sexual dimorphism of cancers and cancer incidences between two countries provide novel model systems for us to reveal the mechanisms underlying sexual dimorphism of human cancers. It would also be worthwhile to investigate whether expression of sex hormone receptors or activity of sex hormone signaling could have geographic differences and how genetic, dietary, and environmental factors could contribute to sexual dimorphism of human cancers, which requires international collaborations on this topic.

The origins of sexual dimorphism are the differences in sex chromosomes in the cells between males and females. The final answers to sexual dimorphism in human cancers could be fully revealed once we have better understanding of how X and Y chromosomes regulate sexual dimorphism?

## Conclusion

We found that most of human cancers have sexual dimorphism in their incidences. Fully understanding the mechanisms underlying sexual dimorphism in human cancers would benefit both basic cancer research and translational application for sex-specific diagnosis, prognosis, and treatment of human cancers, which would be critical for personalized precision medicine.

## Additional file

Additional file 1: **Table S1.** Comparison of incidence rates between men and women in human cancers based on SEER data from 1975 to 2015. **Table S2.** Comparison of incidence rates between men and women in human cancers based on Swedish data from 1975 to 2015. **Table S3.** Comparison of registry cases between men and women in human cancers based on Mayo Clinic data from 1970 to 2015. **Figure S1.** The yearly ratios of men to women case numbers of cancer patients in Mayo Clinic hospitals in 1970–2015. **Figure S2.** The yearly case numbers of cancer patients in Mayo Clinic hospitals in 1970–2015. Blue line, men; red line, women. (PDF 5513 kb)

## Data Availability

The incidence data for all types of cancers were collected from four sources, the SEER Program (NCI/NIH), the cancer incidence data of Sweden (Swedish Cancer Registry), the Cancer Registry data from Mayo Clinic, and the World Cancer Report data in 2012 (IARC). All data and materials are available for other researchers.

## Abbreviations

ASR: Age-standardized rate
NCI: National Cancer Institute
SEER: Surveillance, Epidemiology, and End Results Program
