# Supplementary material for: Sexual dimorphism in the incidence of human cancers
Source: BMC Cancer. 2019 Jul 12;19:684. doi: 10.1186/s12885-019-5902-z (PMC6625025; doi:10.1186/s12885-019-5902-z)
Supplement: Supplementary file 1 — Table S1. Comparison of incidence rates between men and women in human cancers based on SEER data from 1975 to 2015. Table S2. Comparison of incidence rates between men and women in human cancers based on Swedish data from 1975 to 2015. Table S3. Comparison of registry cases between men and women in human cancers based on Mayo Clinic data from 1970 to 2015. Figure S1. The yearly ratios of men to women case numbers of cancer patients in Mayo Clinic hospitals in 1970–2015. Figure S2. The yearly case numbers of cancer patients in Mayo Clinic hospitals in 1970–2015. Blue line, men; red line, women. (PDF 5513 kb) [file 12885_2019_5902_MOESM1_ESM.pdf]

**Table S1** Comparison of incidence rates between men and women in human cancers based on SEER data from 1975 to 2015.

| ID | Cancer Type | Men* (Mean) | Women* (Mean) | Ratio (Mean) | Ratio (Max) | Ratio (Avg) | P-Value (Gamma) | P-Value (Poisson) | P-Value (ANOVA) | P-Value (T-Test) | Category |
|---|---|---|---|---|---|---|---|---|---|---|---|
| 1 | Kaposi Sarcoma | 2.77 | 0.05 | 52.63 | 100.21 | 35.79 | 1.11E-05 | 3.87E-45 | 4.32E-08 | 3.27E-07 | |
| 2 | Cancer of the Larynx | 5.99 | 1.21 | 4.93 | 6.84 | 4.93 | 1.44E-17 | 1.15E-41 | 1.34E-32 | 5.40E-25 | |
| 3 | Mesothelioma | 1.23 | 0.28 | 4.34 | 6.17 | 4.38 | 6.39E-15 | 2.66E-52 | 5.98E-43 | 4.74E-29 | |
| 4 | Urinary Bladder Cancer | 25.12 | 6.48 | 3.88 | 4.27 | 3.88 | 3.34E-16 | 9.52E-57 | 8.49E-88 | 4.11E-54 | |
| 5 | Esophageal Cancer | 5.62 | 1.51 | 3.73 | 4.94 | 3.78 | 4.88E-09 | 3.70E-62 | 2.18E-70 | 2.06E-40 | |
| 6 | Liver Cancer | 6.01 | 2.11 | 2.85 | 3.24 | 2.79 | 9.00E-20 | 1.80E-27 | 1.13E-13 | 4.58E-16 | |
| 7 | Oral Cavity and Pharynx | 14.11 | 5.46 | 2.59 | 2.91 | 2.59 | 2.23E-15 | 2.86E-40 | 1.08E-50 | 2.02E-38 | |
| 8 | Stomach Cancer | 9.09 | 4.18 | 2.18 | 2.48 | 2.17 | 4.90E-18 | 4.20E-30 | 3.37E-27 | 5.20E-27 | |
| 9 | Cancer of the Kidney and Renal Pelvis | 12.34 | 6.10 | 2.02 | 2.40 | 2.04 | 3.03E-16 | 7.68E-26 | 1.32E-20 | 2.93E-29 | |
| 10 | Thyroid Cancer | 3.71 | 10.62 | -2.86 | -2.17 | -2.78 | 7.53E-19 | 7.14E-29 | 3.70E-12 | 1.13E-14 | |
| 11 | Chronic Lymphocytic Leukemia | 4.64 | 2.37 | 1.96 | 2.31 | 1.96 | 2.88E-10 | 1.81E-31 | 1.28E-42 | 6.15E-38 | |
| 12 | Non-Small Cell Lung Cancer | 53.60 | 28.48 | 1.88 | 3.60 | 1.98 | 1.68E-07 | 1.28E-06 | 1.56E-26 | 3.40E-16 | Sex-dimorphic |
| 13 | Chronic Myeloid Leukemia | 1.74 | 1.04 | 1.68 | 2.12 | 1.69 | 3.12E-06 | 3.71E-15 | 3.94E-41 | 9.76E-28 | |
| 14 | Small Cell lung Cancer | 8.48 | 5.44 | 1.56 | 2.73 | 1.59 | 3.04E-10 | 4.76E-13 | 1.07E-10 | 3.72E-11 | |
| 15 | Rectal Cancer | 10.00 | 5.90 | 1.70 | 1.90 | 1.70 | 4.81E-13 | 9.36E-35 | 2.91E-36 | 9.69E-35 | |
| 16 | Myeloma | 5.13 | 3.43 | 1.50 | 1.71 | 1.50 | 3.63E-11 | 7.29E-26 | 6.36E-25 | 2.25E-28 | |
| 17 | Acute Myeloid Leukemia | 3.26 | 2.32 | 1.40 | 1.67 | 1.41 | 7.98E-03 | 1.26E-22 | 1.08E-22 | 2.96E-29 | |
| 18 | Non-Hodgkin Lymphoma | 16.35 | 11.00 | 1.49 | 1.69 | 1.48 | 2.05E-14 | 3.77E-20 | 1.87E-14 | 4.00E-24 | |
| 19 | Brain Cancer | 6.37 | 4.46 | 1.43 | 1.56 | 1.43 | 7.68E-04 | 2.72E-32 | 3.77E-47 | 5.33E-36 | |
| 20 | Melanoma of the Skin | 15.82 | 11.88 | 1.33 | 1.46 | 1.31 | 1.58E-18 | 1.07E-18 | 6.83E-05 | 3.41E-15 | |
| 21 | Acute Lymphocytic Leukemia | 1.69 | 1.29 | 1.32 | 1.70 | 1.33 | 8.12E-01 | 8.03E-10 | 5.92E-13 | 8.71E-19 | |
| 22 | Hodgkin Lymphoma | 3.18 | 2.45 | 1.30 | 1.79 | 1.31 | 1.34E-03 | 5.16E-35 | 4.70E-26 | 4.31E-18 | |
| 23 | Pancreatic Cancer | 9.68 | 7.14 | 1.36 | 1.72 | 1.36 | 1.01E-02 | 9.92E-29 | 4.67E-37 | 1.66E-28 | |
| 24 | Colon Cancer | 30.51 | 24.17 | 1.26 | 1.38 | 1.26 | 4.80E-11 | 7.29E-12 | 8.85E-09 | 2.41E-22 | |
| 25 | Breast Cancer | 0.78 | 98.66 | -125.74 | -156.98 | -126.49 | 1.07E-19 | 2.54E-64 | 9.54E-71 | 3.81E-42 | |
| 26 | Cancer of the Cervix Uteri | - | 8.03 | - | - | - | - | - | - | - | |
| 27 | Cancer of the Corpus and Uterus, NOS | - | 21.12 | - | - | - | - | - | - | - | Female-specific |
| 28 | Ovarian Cancer | - | 11.43 | - | - | - | - | - | - | - | |
| 29 | Prostate Cancer | 103.45 | - | - | - | - | - | - | - | - | Male-specific |
| 30 | Testis Cancer | 5.22 | - | - | - | - | - | - | - | - | |

*, The incidence rates are per 100,000 and are age-adjusted to the World Standard population (WHO 2000-2025). Ratio, men/women. Max, maximum ratio; Avg, average. -, data not available.

**Table S2** Comparison of incidence rates between men and women in human cancers based on Swedish data from 1975 to 2015.

| ID | Cancer Type | Men* (Mean) | Women* (Mean) | Ratio (Mean) | Ratio (Max) | Ratio (Avg) | P-Value (Gamma) | P-Value (Poisson) | P-Value (ANOVA) | P-Value (T-Test) | Comment |
|---|---|---|---|---|---|---|---|---|---|---|---|
| 1 | Kaposi Sarcoma | 0.40 | 0.09 | 4.44 | 24.54 | 5.54 | 2.28E-15 | 0.00E+00 | 4.40E-15 | 1.09E-12 | |
| 2 | Cancer of the Larynx | 2.53 | 0.36 | 7.11 | 18.65 | 7.58 | 9.87E-17 | 3.84E-60 | 2.40E-43 | 2.35E-28 | |
| 3 | Mesothelioma | 1.06 | 0.19 | 5.49 | 13.26 | 5.93 | 9.63E-18 | 1.32E-77 | 2.12E-29 | 6.12E-23 | |
| 4 | Urinary Bladder Cancer | 19.50 | 5.43 | 3.59 | 4.33 | 3.59 | 1.37E-19 | 1.41E-43 | 1.14E-61 | 1.07E-42 | |
| 5 | Esophageal Cancer | 3.61 | 1.14 | 3.17 | 4.08 | 3.22 | 1.12E-09 | 9.26E-43 | 7.33E-69 | 3.04E-42 | |
| 6 | Liver Cancer | 5.62 | 5.03 | 1.12 | 1.83 | 1.18 | 8.55E-10 | 3.12E-05 | 2.94E-02 | 1.16E-04 | |
| 7 | Oral Cavity and Pharynx | 7.17 | 3.52 | 2.03 | 3.19 | 2.11 | 6.66E-04 | 2.29E-21 | 5.50E-45 | 1.43E-31 | |
| 8 | Stomach Cancer | 12.55 | 6.47 | 1.94 | 2.28 | 1.93 | 5.78E-21 | 2.68E-23 | 4.85E-09 | 2.18E-18 | |
| 9 | Cancer of the Kidney and Renal Pelvis | 10.55 | 6.23 | 1.69 | 1.96 | 1.69 | 1.17E-11 | 3.35E-30 | 1.59E-31 | 1.77E-34 | |
| 10 | Thyroid Cancer | 1.74 | 4.53 | -2.61 | -3.32 | -2.61 | 1.91E-18 | 1.20E-32 | 2.27E-26 | 4.25E-25 | |
| 11 | Chronic Lymphocytic Leukemia | 3.45 | 1.72 | 2.01 | 2.75 | 2.03 | 1.39E-08 | 6.99E-31 | 2.36E-38 | 4.44E-36 | |
| 12 | Non-Small Cell Lung Cancer | 22.42 | 11.23 | 2.00 | 4.39 | 2.34 | 3.03E-01 | 1.29E-02 | 3.19E-23 | 2.00E-13 | Sex-dimorphic |
| 13 | Chronic Myeloid Leukemia | 1.01 | 0.70 | 1.44 | 2.19 | 1.48 | 2.54E-04 | 1.10E-29 | 3.82E-11 | 3.31E-12 | |
| | Small Cell lung Cancer | 2.36 | 1.60 | 1.48 | 4.68 | 1.52 | 2.70E-03 | 7.47E-09 | 2.15E-02 | 1.47E-05 | |
| 14 | Rectal Cancer | 13.59 | 8.46 | 1.61 | 1.84 | 1.61 | 8.54E-05 | 1.50E-37 | 1.92E-56 | 1.14E-41 | |
| 15 | Myeloma | 4.25 | 2.87 | 1.48 | 1.83 | 1.49 | 5.45E-04 | 1.54E-35 | 2.83E-44 | 2.99E-29 | |
| 16 | Acute Myeloid Leukemia | 2.07 | 1.76 | 1.17 | 1.57 | 1.19 | 9.74E-01 | 1.99E-17 | 2.55E-08 | 1.16E-11 | |
| 17 | Non-Hodgkin Lymphoma | 8.17 | 5.54 | 1.47 | 2.03 | 1.53 | 5.13E-15 | 9.49E-11 | 6.75E-05 | 3.74E-22 | |
| 18 | Brain Cancer | 0.99 | 0.88 | 1.13 | 1.60 | 1.17 | 3.75E-01 | 1.04E-17 | 1.48E-04 | 2.07E-04 | |
| 19 | Melanoma of the Skin | 12.76 | 13.14 | 0.97 | 1.12 | 0.96 | 7.75E-02 | 8.13E-01 | 7.31E-01 | 1.23E-03 | |
| 20 | Acute Lymphocytic Leukemia | 1.56 | 1.27 | 1.24 | 1.88 | 1.27 | 3.36E-01 | 1.34E-05 | 2.28E-07 | 5.98E-09 | |
| 21 | Hodgkin Lymphoma | 2.44 | 1.77 | 1.38 | 2.17 | 1.39 | 2.55E-12 | 4.25E-19 | 1.23E-11 | 1.41E-11 | |
| 22 | Pancreatic Cancer | 7.94 | 6.29 | 1.26 | 1.66 | 1.25 | 3.22E-16 | 3.53E-16 | 4.66E-07 | 6.36E-13 | |
| 23 | Colon Cancer | 21.44 | 18.93 | 1.13 | 1.26 | 1.13 | 9.39E-08 | 3.15E-13 | 2.11E-12 | 1.30E-21 | |
| 24 | Breast Cancer | 0.51 | 83.08 | -164.45 | -237.15 | -168.40 | 5.49E-24 | 1.04E-73 | 1.52E-51 | 5.99E-33 | |
| 25 | Cancer of the Cervix Uteri | - | 9.72 | - | - | - | - | - | - | - | |
| 26 | Cancer of the Corpus and Uterus, NOS | - | 14.79 | - | - | - | - | - | - | - | Female-specific |
| 27 | Ovarian Cancer | - | 13.26 | - | - | - | - | - | - | - | |
| 28 | Prostate Cancer | 81.43 | - | - | - | - | - | - | - | - | Male-specific |
| 29 | Testis Cancer | 5.35 | - | - | - | - | - | - | - | - | |

*, The incidence rates are per 100,000 and are age-adjusted to the World Standard population (WHO 2000-2025).. Ratio, men/women. Max, maximum ratio; Avg, average. -, data not available.

**Table S3** Comparison of registry cases between men and women in human cancers based on Mayo Clinic data from 1970 to 2015.

| ID | Cancer Type | Men* (Mean) | Women* (Mean) | Ratio (Mean) | Ratio (Max) | Ratio (Avg) | P-Value (Gamma) | P-Value (Poisson) | P-Value (ANOVA) | P-Value (T-Test) | Comment |
|---|---|---|---|---|---|---|---|---|---|---|---|
| 1 | Kaposi Sarcoma | 3.76 | 0.71 | 5.28 | 8.00 | 2.79 | - | 1.19E-11 | 3.73E-14 | 5.22E-13 | Sex-dimorphic |
| 2 | Cancer of the Larynx | 85.57 | 15.96 | 5.36 | 14.60 | 6.38 | 4.08E-12 | 1.69E-04 | 6.73E-38 | 9.83E-31 | |
| 3 | Mesothelioma | 30.46 | 10.26 | 2.97 | 16.00 | 3.54 | 1.01E-12 | 7.34E-07 | 5.85E-11 | 4.89E-19 | |
| 4 | Urinary Bladder Cancer | 345.93 | 83.83 | 4.13 | 6.32 | 4.25 | 1.12E-18 | 2.09E-02 | 3.37E-18 | 2.83E-15 | |
| 5 | Esophageal Cancer | 133.35 | 32.65 | 4.08 | 6.89 | 3.72 | 7.13E-18 | 2.85E-03 | 5.97E-08 | 1.07E-09 | |
| 6 | Liver Cancer | 122.48 | 59.33 | 2.06 | 4.50 | 1.95 | 9.34E-12 | 2.19E-02 | 1.92E-03 | 9.21E-08 | |
| 7 | Oral Cavity and Pharynx | 216.65 | 112.89 | 1.92 | 2.60 | 1.86 | 6.50E-19 | 1.70E-04 | 1.23E-08 | 4.55E-14 | |
| 8 | Stomach Cancer | 137.15 | 64.76 | 2.12 | 4.41 | 2.32 | 1.42E-07 | 2.88E-01 | 3.23E-17 | 9.97E-23 | |
| 9 | Cancer of the Kidney and Renal Pelvis | 260.33 | 118.74 | 2.19 | 2.74 | 2.17 | 6.46E-18 | 1.25E-02 | 5.93E-06 | 4.52E-12 | |
| 10 | Thyroid Cancer | 88.02 | 151.48 | -1.72 | -2.88 | -1.81 | 5.28E-16 | 3.50E-02 | 2.50E-03 | 6.39E-08 | |
| 11 | Chronic Lymphocytic Leukemia | 115.96 | 58.84 | 1.97 | 2.90 | 2.01 | 1.24E-15 | 2.70E-01 | 7.34E-07 | 5.69E-15 | |
| 12 | Non-Small Cell Lung Cancer | 543.93 | 354.80 | 1.53 | 4.54 | 1.92 | 8.44E-01 | 1.78E-01 | 8.86E-05 | 3.56E-15 | |
| 13 | Chronic Myeloid Leukemia | 24.83 | 18.09 | 1.37 | 5.00 | 1.47 | 1.36E-07 | 7.36E-02 | 1.43E-02 | 1.58E-08 | |
| 14 | Small Cell lung Cancer | 73.63 | 48.43 | 1.52 | 7.10 | 1.90 | 6.08E-02 | 4.44E-02 | 1.95E-06 | 4.82E-11 | |
| 15 | Rectal Cancer | 169.30 | 102.15 | 1.66 | 2.25 | 1.66 | 1.59E-14 | 5.05E-04 | 5.86E-09 | 2.47E-18 | |
| 16 | Myeloma | 148.07 | 103.26 | 1.43 | 2.14 | 1.50 | 6.55E-16 | 5.87E-01 | 3.48E-02 | 1.07E-10 | |
| 17 | Acute Myeloid Leukemia | 58.11 | 40.22 | 1.44 | 4.60 | 1.58 | 3.18E-12 | 1.72E-01 | 2.72E-02 | 3.55E-11 | |
| 18 | Non-Hodgkin Lymphoma | 257.63 | 193.59 | 1.33 | 1.80 | 1.31 | 1.04E-14 | 9.16E-01 | 6.22E-02 | 3.55E-10 | |
| 19 | Brain Cancer | 272.11 | 270.39 | 1.01 | 1.56 | 1.05 | 8.02E-05 | 3.04E-01 | 9.50E-01 | 7.29E-01 | |
| 20 | Melanoma of the Skin | 287.52 | 176.13 | 1.63 | 2.20 | 1.48 | 8.10E-17 | 2.24E-01 | 7.68E-03 | 8.18E-08 | |
| 21 | Acute Lymphocytic Leukemia | 15.96 | 10.69 | 1.49 | 10.00 | 1.73 | 3.78E-04 | 1.55E-01 | 2.36E-04 | 1.23E-04 | |
| 22 | Hodgkin Lymphoma | 51.63 | 40.30 | 1.28 | 2.54 | 1.35 | 3.50E-03 | 6.41E-01 | 1.12E-03 | 9.77E-09 | |
| 23 | Pancreatic Cancer | 137.68 | 117.96 | 1.17 | 1.84 | 1.16 | 3.62E-03 | 8.24E-01 | 5.10E-01 | 5.10E-01 | |
| 24 | Colon Cancer | 338.52 | 287.65 | 1.18 | 1.46 | 1.19 | 1.56E-02 | 4.27E-01 | 1.66E-02 | 2.12E-15 | |
| 25 | Breast Cancer | 9.63 | 1212.35 | -125.89 | -352.50 | -139.11 | 2.18E-20 | 1.02E-12 | 2.15E-24 | 3.62E-18 | Female-specific |
| 26 | Cancer of the Cervix Uteri | - | 121.59 | - | - | - | - | - | - | - | |
| 27 | Cancer of the Corpus and Uterus, NOS | - | 224.02 | - | - | - | - | - | - | - | |
| 28 | Ovarian Cancer | - | 181.13 | - | - | - | - | - | - | - | |
| 29 | Prostate Cancer | 1648.61 | - | - | - | - | - | - | - | - | Male-specific |
| 30 | Testis Cancer | 51.54 | - | - | - | - | - | - | - | - | |

*, The cancer registry cases from Mayo Clinic include all clinical records of cancer patients at Mayo Clinic Hospitals for all three sites in the country, Rochester, MN, Scottsdale, AZ, and Jacksonville, FL. Ratio, men/women. Max, maximum ratio; Avg, average. -, data not available.

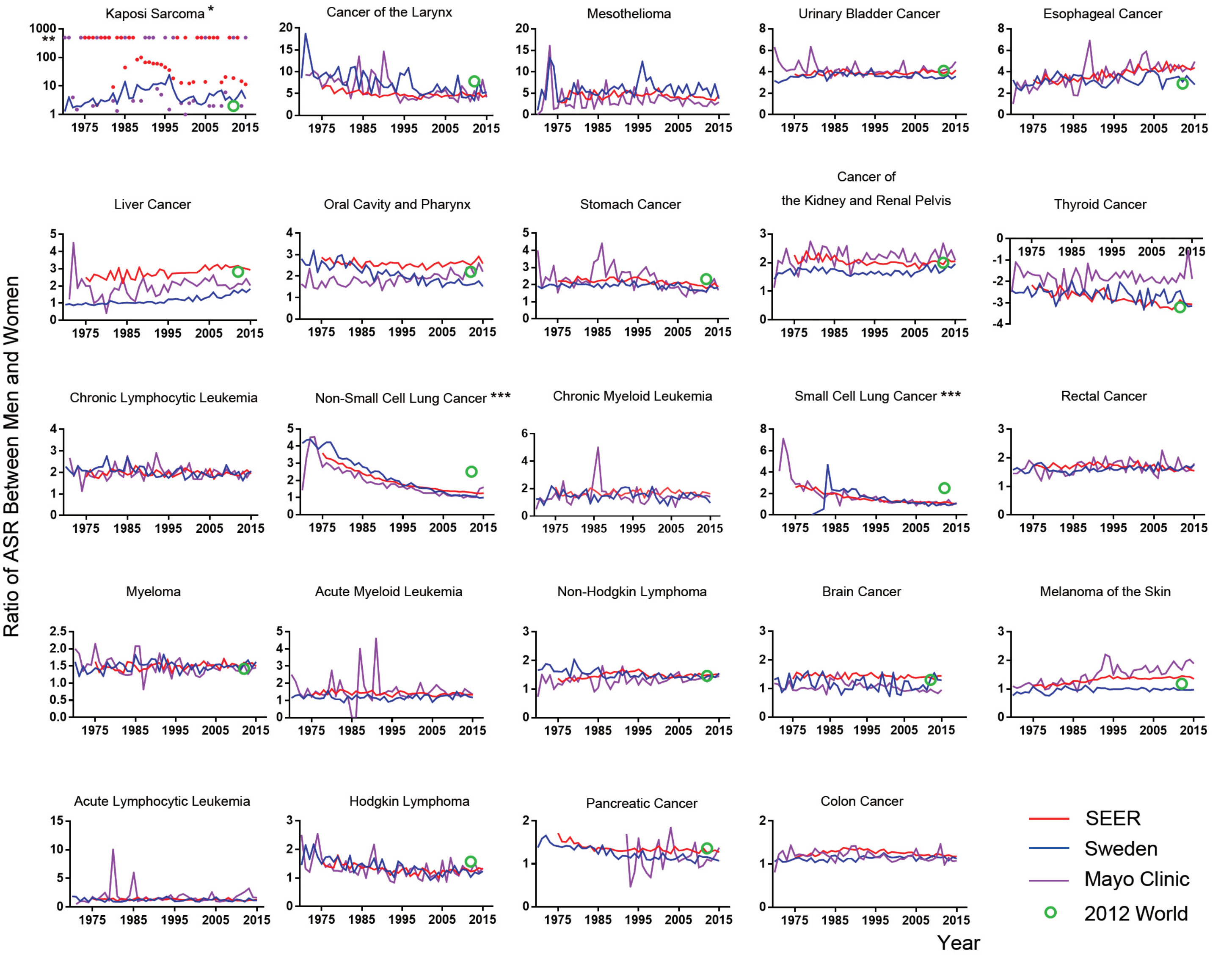
Kaposi Sarcoma *
Cancer of the Larynx
Mesothelioma
Urinary Bladder Cancer
Esophageal Cancer
Liver Cancer
Oral Cavity and Pharynx
Stomach Cancer
Cancer of the Kidney and Renal Pelvis
Thyroid Cancer
Chronic Lymphocytic Leukemia
Non-Small Cell Lung Cancer ***
Chronic Myeloid Leukemia
Small Cell Lung Cancer ***
Rectal Cancer
Myeloma
Acute Myeloid Leukemia
Non-Hodgkin Lymphoma
Brain Cancer
Melanoma of the Skin
Acute Lymphocytic Leukemia
Hodgkin Lymphoma
Pancreatic Cancer
Colon Cancer
Ratio of ASR Between Men and Women
Year
SEER
Sweden
Mayo Clinic
2012 World

Figure s1

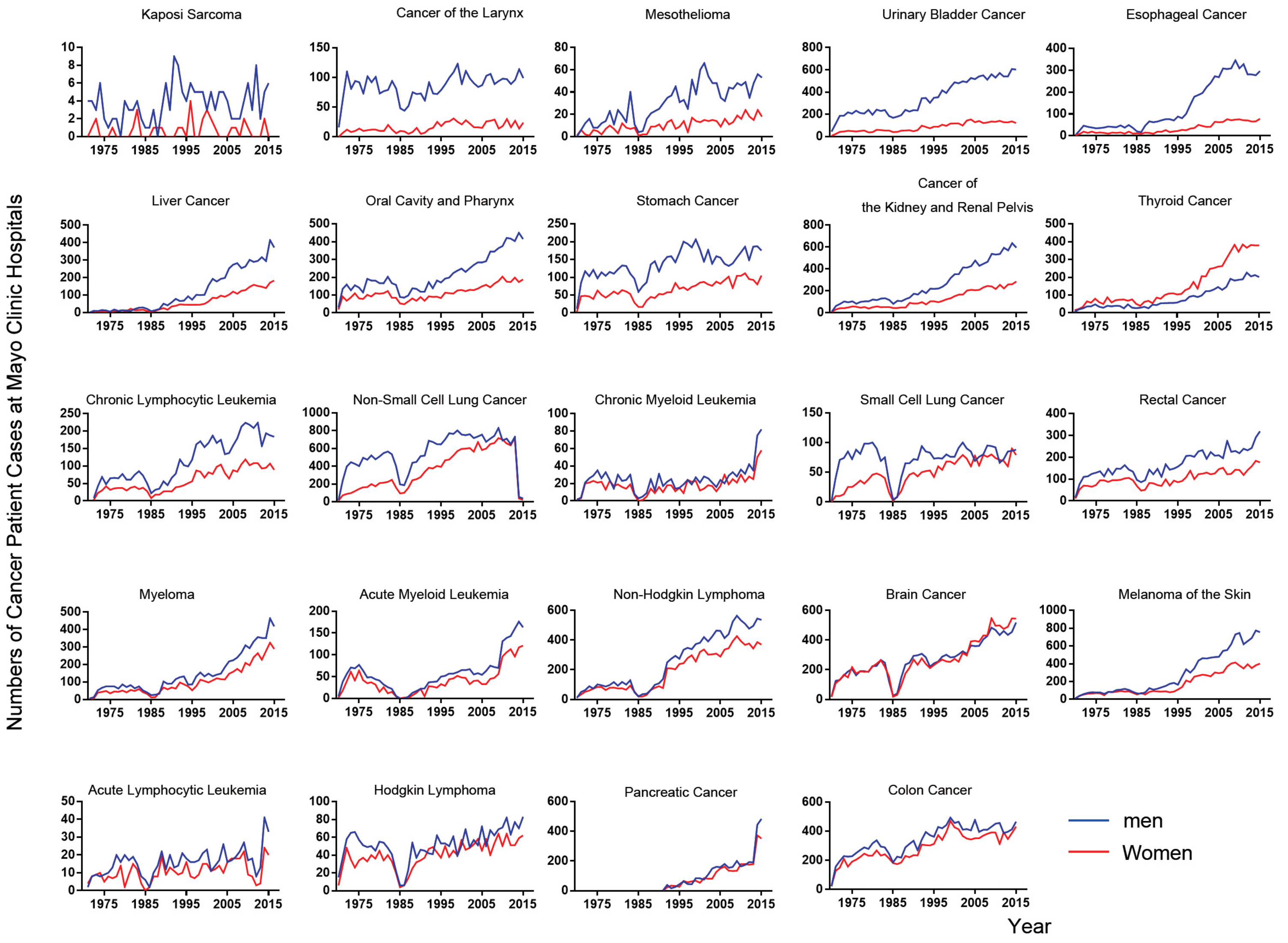

Numbers of Cancer Patient Cases at Mayo Clinic Hospitals
Kaposi Sarcoma
Cancer of the Larynx
Mesothelioma
Urinary Bladder Cancer
Esophageal Cancer
Liver Cancer
Oral Cavity and Pharynx
Stomach Cancer
Cancer of
the Kidney and Renal Pelvis
Thyroid Cancer
Chronic Lymphocytic Leukemia
Non-Small Cell Lung Cancer
Chronic Myeloid Leukemia
Small Cell Lung Cancer
Rectal Cancer
Myeloma
Acute Myeloid Leukemia
Non-Hodgkin Lymphoma
Brain Cancer
Melanoma of the Skin
Acute Lymphocytic Leukemia
Hodgkin Lymphoma
Pancreatic Cancer
Colon Cancer
men
Women
Year


Figure S2
